# Discussion on the validity of commonly used reliability indices in sports medicine and exercise science: a critical review with data simulations

**DOI:** 10.1007/s00421-025-05720-6

**Published:** 2025-02-13

**Authors:** Konstantin Warneke, Thomas Gronwald, Sebastian Wallot, Alessia Magno, Martin Hillebrecht, Klaus Wirth

**Affiliations:** 1 Institute for Sustainability Education and Psychology, Leuphana University of Lüneburg, Lüneburg, Germany; 2 Institute of Human Movement Science, Sport and Health, University of Graz, Graz, Austria; 3 Institute of Interdisciplinary Exercise Science and Sports Medicine, MSH Medical School Hamburg, Hamburg, Germany; 4 G-Lab, Faculty of Applied Sport Sciences and Personality, BSP Business and Law School, Berlin, Germany; 5 Faculty of Kinesiology and Physical Education, University of Toronto, Toronto, Canada; 6 University Sports Center, Carl Von Ossietzky University Oldenburg, Oldenburg, Germany; 7 Department of Training and Sport, University of Applied Sciences Wiener Neustadt, Wiener Neustadt, Austria

**Keywords:** Practical relevance, Precision, Accuracy, Systematic errors, Random errors

## Abstract

Apart from objectivity and validity, reliability is considered a precondition for testing within scientific works, as unreliable testing protocols limit conclusions, especially for practical application. Classification guidelines commonly refer to relative reliability, focusing on Pearson correlation coefficients (*r*_p_) and intraclass correlation coefficients (ICC). On those, the standard error of measurement (SEM) and the minimal detectable change (MDC) are often calculated in addition to the variability coefficient (CV). These, however, do not account for systematic or random errors (e.g., standardization problems). To illustrate, we applied common reliability statistics in sports science on simulated data which extended the sample size of two original counter-movement-jump sessions from (youth) elite basketball players. These show that excellent *r*_p_ and ICC (≥ 0.9) without a systematic bias were accompanied by a mean absolute percentage error of over 20%. Furthermore, we showed that the ICC does not account for systematic errors and has only limited value for accuracy, which can cause misleading conclusions of data. While a simple re-organization of data caused an improvement in relative reliability and reduced limits of agreement meaningfully, systematic errors occurred. This example underlines the lack of validity and objectivity of commonly used ICC-based reliability statistics (SEM, MDC) to quantify the primary and secondary variance sources. After revealing several caveats in the literature (e.g., neglecting of the systematic and random error or not distinguishing between protocol and device reliability), we suggest a methodological approach to provide reliable data collections as a precondition for valid conclusions by, e.g., recommending pre-set acceptable measurement errors.

**Supplementary Information:**

The online version contains supplementary material available at 10.1007/s00421-025-05720-6.

## Introduction

The athlete’s or patients’ health and well-being as well as the individual level of capacity and performance is the center of the trainers and therapists’ effort. Independent of the exact aim (rehabilitation, sports performance etc.), it is well accepted that evidence-based practice seems to be the gold standard (Riemann and Lininger 2015). To prove this paradigm, evidence should be produced, and recommendations derived based on structured data collection and appropriate statistical analysis to form high-quality foundation of practical applications (French and Torres Ronda 2022). As a consequence, developing an objective, and well-balanced perspective is assumed to be essential in “facilitating ongoing training and performance interventions”, which is the sport scientists’ main task (French and Torres Ronda 2022). For appropriate implementation, an adequate and accurate data collection is considered the center of generating evidence (Currell and Jeukendrup 2008; Hopkins 2000). To reach maximal standards, the scientific community agreed on criteria such as objectivity, reliability and validity (Currell and Jeukendrup 2008) with the aim to adhere to accuracy, precision and reproducibility. Accordingly, while these criteria are just one piece of a larger (statistical) puzzle including, for instance, a prior sample size estimation, power analyses and *p* value adjustments, reliability and repeatability can be considered mandatory for further data processing and interpretation of results (Hopkins 2000). In turn, when practitioners review longitudinal and experimental studies to implement innovative and new training routines (Lamb 1998), they should trust that data collection was performed (at least) under reliable and objective conditions, as unreliable data cannot bring valid results (what does not automatically mean that every reliable data collection result in valid results).

While objectivity refers to testing protocols leading to the same result independent of the assessor, Atkinson and Nevill (1998) described validity as the ability of the testing device to measure the actual value, which might be assessed via the agreement between the observed value and the true criterion value investigated via the used diagnostical procedure (Hopkins 2000; Bland and Altmann 1986). Even though both criteria might be of relevance, Hopkins (2000) and Atkinson and Nevill (1998) stressed the paramount importance of reliable testing procedures as those that provide essential information about measurement errors, arising from repeating the measurement. However, when does a measurement protocol fulfill the required standards of objectivity, validity, and reliability, and how is a satisfactory level reached? While several reliability indices exist in the literature and are commonly applied to justify the reliability of the performed measurement, the transparent quantification of measurement errors arises from repeating the testing in a comprehensive way. This transparent and detailed quantification is in the responsibility of scientists, and not the task of practitioners and clinicians. Otherwise, misinterpretations can cause clinical decision errors creating substantial impact on the health and well-being of patients or athletes (Charter 2003).

In the present work, the focus is on the test–retest reliability problem and to critically discuss the current way of dealing with frequently stated key parameters to classify reliability as satisfying. Therefore, the most frequently used reliability indices in sport and exercise science literature in the light of the original aim to assess reliability are introduced in the first step. Afterwards, these are applied to a simulated data set to illustrate limitations and weaknesses of current interpretations with their subsequent recommendations for practical and clinical usage, with special consideration of systematic and random errors (Hopkins 2000; Lamb 1998; Atkinson and Nevill 1998; Vetter and Schober 2018). Therefore, with this work, higher awareness for appropriate measurement error quantification should be raised, which are necessary to transparently report results and improve interpretability of those in the sports and exercise science community. These arguments will then be used to develop a guideline to appropriately collect data and how to minimize or, at least, quantify measurement errors for subsequent study protocols.

## What we aim to quantify—and what we actually do

When performing measurements in humans, the occurrence of measurement errors is inherent (Barnhart et al. 2007). One source of those errors arises from multiple and repeated testing, which directly affects the certainty of evidence resulting from the study (Atkinson and Nevill 1998) with direct implications for evidence-based recommendations for practitioners and clinicians (Riemann and Lininger 2015). Therefore, an overall understanding of reliability seems mandatory.

There are several definitions of reliability, which have partially changed over time. The most intuitive definition might describe a reliable measurement to produce the same result when measuring multiple times in a row (Atkinson and Nevill 1998). Originally defined as the ratio of a true score variance to the observed total score variance (Cronbach et al. 1972; Lord and Novick 1986), Hopkins (2000) described retest reliability to concern the reproducibility of the observed value when repeating the measurement. Barnhart et al. (2007) dated their definition back to the *Food and Drug Administrations (FDA) guidelines on bioanalytical validations* (Food and Drug Administration 2001) as well as the *principles of accuracy of measurement* guidelines provided by the International Organization for Standardization (ISO) from 1994, which were recently updated (International Organization of Standardization 2023). They distinguished between repeatability and reproducibility as two distinct types of precision under extreme conditions. Thus, and of high interest for the further discussion, precision is defined as the closeness of agreement, described as the degree of scatter between a series of measurements from multiple testing time points of the same homogenous sample under prescribed conditions. Accordingly, the ISO definition is similar and described precision as the closeness of agreement between independent test results obtained under stipulated conditions. Repeatability was described as the agreement between independent test results under constant conditions.

Assuming a testing procedure to be reliable, it is important to produce independent test results with the same method in identical participants in the same laboratory from the same assessor operating, with the same equipment and under the same environmental conditions. This can be quantified for one testing session (*intraday*) or between testing sessions (*interday*) (Barnhart et al. 2007). Indeed, repeatability should produce minimal deviations in the results, which can be described as high intraday reliability, referring to the extent to which a test provides a measurement value that is free of error over repeated trials (Riemann and Lininger 2015). While a brief overview of the differences is provided in Table S1 in the Supplemental Material, the concept of reliability, i.e., repeatability can be easily illustrated by an example from practical performance testing which will be discussed throughout this study. One frequently performed test to assess explosive strength capabilities are vertical jumping tests. It seems obvious that the testing procedure matched reliability criteria if the jumping height performance is tested twice and produces the exact same results (for example: Test 1: 40 cm, Test 2: 40 cm). However, in practice and research, there is not just one athlete. When testing several participants, not all of them will reach the exact same jumping height in their second trial. This deviation must be adequately assessed, quantified and reported to enable a viable interpretation of the study results under consideration of different influencing factors.

To quantify and subsequently classify deviations between the results, for example directly following trials in the testing population, there are several indices used in sports and exercise science to explore the performed testing under reliable conditions.

## Most frequently used metrics in sports and exercise science and their classification

First reliability quantification approaches were provided by Galton (1886) as well as Pearson (1986) and Fisher (1925) from biological, and medical sciences, leading to first intraclass correlations (ICC) to measure reliability. With an increasing number of measurement methods and testing procedures, the reliability approaches were further developed to generalize the results (Cronbach et al. 1972; Vangeneugden et al. 2005), which causes several reliability indices with diverse classifications. The most common way to report reliability is to provide correlation-based reliability statistics. While it is usual to report Pearson’s* r* (*r*_p_), the two-way mixed effects model ICC for absolute agreement or consistency (Koo and Li 2016) is currently the most common metric. Some authors supplement these with the coefficient of variability (CV), defined as the ratio of the standard deviation and the mean, multiplied by 100 (Shechtman et al. 2013; Anvari et al. 2018). The CV might provide the advantage of a dimensionless coefficient for proportional changes of the means and standard deviations (Reed et al. 2002; Kottner et al. 2011). It is noteworthy that both r_p_ and ICC assume a linear relationship between the included variables, while the ICC provides a more detailed evaluation by accounting for the bias degree between the raters or inserted values (Vetter and Schober 2018).

Anvary et al. (2018) described an ICC of 1.0 to represent perfect reliability. If the measurement error is small relative to variability between participants, the ICC approaches 1.0. While an ICC of 0.7 is frequently classified as large, thus good, Koo and Li (2016) suggested a threshold of 0.9. Considering results of Kottner et al. (2011) and Fleiss (1999), Anvari et al. (2018) preferred an ICC of 0.95 to be associated with an acceptable measurement error for most measurements in sports science and medicine. However, the question arises about the justification to classify a reliability value to be sufficient or not (Atkinson and Nevill 1998).

To facilitate the interpretation, several authors added the standard error of measurement (SEM) (Geerinck et al. 2019; Palta et al. 2011; Tighe et al. 2010) to receive a reliability parameter in the same dimension of the measured value. As mentioned above, in longitudinal study designs, reliability might have some influence on the certainty of found results. A pre–post-change might be hardly interpretable if the measurement error resulting from reliability surpasses the expected or measured intervention induced change. With the aim to counteract this concern and to reach an appropriate pre–post-change higher than the measurement error to assist practitioners in decision making (Paraskevopoulos et al. 2023), some authors calculated the minimal detectable change (MDC) (Steffen and Seney 2008; Powden et al. 2015; Seamon et al. 2022).

As early as 1998, Lamb (1998) described measurement concepts in exercise science and pointed out the necessity of distinguishing between relative and absolute reliability as subsequent errors originate from different sources. Internal consistency reliability is described as the variability between repeated trials within a day (Atkinson and Nevill 1998), while stability reliability can be considered equal to repeatability (Barnhart et al. 2007). Interestingly, the SEM is commonly calculated to account for absolute reliability statements but fails in addressing the measurement error objectively (Atkinson and Nevill 1998; Roebroeck et al. 1993).

## Relative reliability indices versus systematic and random measurement error quantification

When assessing reliability, several sources of measurement errors must be acknowledged. The systematic measurement error refers to a systematic shift of the mean and standard deviation resulting from a testing procedure (e.g., due to learning effects and fatigue) (Atkinson and Nevill 1998; Barnhart et al. 2007). In addition, Hopkins (2000) described the random error as “noise”, referring to the random scattering of individual deviations, which might be attributable to sources of secondary variance. This error type is also described by the ISO. The authors describe “The need to consider “precision” arises because tests or measures performed on presumably identical test items in presumably identical circumstances do not, in general, yield identical results”. This is attributed to unavoidable random errors inherent in every measurement procedure; the factors that influence the outcome of a measurement cannot all be completely controlled. In the practical interpretation of measurement data, this variability should be taken into account (International Organization of Standardization 2023). Moreover, precision must be differentiated from accuracy. Decreasing the random scattering/secondary variance meaningfully improves precision, but not necessarily accuracy of the test. A perfect example can be learning or warm-up effects in a homogeneous sample. Without warming up, participants can produce maximal strength precisely, meaning that 10 participants who started testing without warming up increase their strength from trial 1 to trial 2 all exactly by 200 N. The testing will show no random error (highly precise), however, it was not accurate to test the maximal strength capacity, as participants showed systematic errors, in our case arising from warming up. Unfortunately, the ICC does not allow distinguishing between and account for both forms of measurement errors, which will be illustrated in examples in the following. This lack of quantification limits the interpretability about precision and accuracy of the measurement (Lamb 1998; International Organization of Standardization 2023). The main limitation of commonly used reliability indices can be considered as a simplification and, therefore, neglection of essential aspects.

Assuming a test to be repeatable, performing the same measurement procedure several times in a row within one session would produce the exact same results, therefore, the measurements would, in fact, agree with each other (high precision). In sports and exercise science, a 100% agreement between different trials seems more theoretical than practically relevant (International Organization of Standardization 2023). The question arises whether the relative reliability indices alone are valid to quantify reliability or, i.e., repeatability and to account for systematic and random errors. As one is interested in receiving information about the deviation between the test and the retest throughout the whole sample, and not on the regression that fits with a minimal distance to the relationship between the test and the retest value (as it is provided via relative reliability indices with [ICC] or without [*r*_p_] an analysis of variance), it seems insufficient to exclusively state relative reliability statistics. To easily illustrate these limitations, the initially stated example of jumping height testing is taken up. Now, imagine athlete A with test/retest jumping height values of 40/35 cm, athlete B with 45/49 cm and athlete C with 36/31 cm, while athletes D and E jump 32/29 cm and 22/18 cm, respectively. Obviously, in all tests, the second jump shows a practically relevant difference to the first jump. Here, *r*_p_ using the following equation can be applied:

1 $$r_{p} \frac{{\mathop \sum \nolimits_{i = 1}^{n} \left( {x_{i} - \overline{x}} \right)\left( {y_{i} - \overline{y}} \right)}}{{\sqrt {\left( {\mathop \sum \nolimits_{i = 1}^{n} \left( {x_{i} - \overline{x}} \right)^{2} } \right) \left( {\mathop \sum \nolimits_{i = 1}^{n} \left( {y_{i} - \overline{y}} \right)^{2} } \right)} }}$$

where *n* is the number of data points, *i* is the index for each (paired) data point, *x*_*i*_ is the *i*-th data point in variable *x* (e.g., jumping height at trial 1), and *y*_*i*_ is the *i*-th data point in variable *y* (e.g., jumping height at trial 2).

This results in a coefficient classified “excellent” (*r*_p_ = 0.96). That correlation coefficients are invalid to determine reliability/agreement/repeatability is no novelty. As early as 1986 and 1989, Bland and Altmann (1986) as well as Lin (1989) described correlations to be a distinctively unequal construct compared to agreement (Bland and Altmann 1986; Vetter and Schober 2018; Lin 1989; Hung et al. 2017). Accordingly, most current studies use one ICC type to justify their study protocol as reliable. Even though several ways to calculate the ICC exist (Weir 2005), their calculation method remind to those of analyses of variance using means and standard deviations. The ICC seems more appropriately measure associations between two values than the Pearson correlation does. Currently, there are three different approaches consisting of one-way random, two-way random and two-way mixed calculation models available (Shrout and Fleiss 1979; McGraw and Wong 1996). However, also for the SEM and the MDC, several classifications, nomenclatures and formulas exist (the MDC is also known as the smallest detectable change or smallest worthwhile difference; Mann et al. 2014), which must be differentiated from the smallest worthwhile change (SWC) which was calculated ${\text{SWC}} = 0.2*{\text{SD }}$ (Hopkins 2004) or minimal difference (Weir 2005). In addition, the SEM is sometimes called typical error (TE) (Mann et al. 2014). To avoid confusion by introducing and mixing up so many metrics, the focus will be on the following and, most common way, for example, the two-way ICC for agreement (Koo and Li 2016).

### Calculation formulas on reliability metrics used in this work

2 $${\text{ ICC}} = {\text{MS}}_{R} - {\text{MS}}_{E} /\left( {{\text{MS}}_{R} + \left( {{\text{MS}}_{C} - {\text{MS}}_{E} } \right)/n} \right)$$

where ICC is the intraclass correlation coefficient, ${\text{MS}}_{C}$ is the mean square for columns, ${\text{MS}}_{E}$ is the mean square for error, ${\text{MS}}_{R}$ is the mean square for rows, and *n* is the number of subjects.

However, for the five participants mentioned above, also the ICC would suggest excellent reliability (ICC for consistency = 0.93, ICC for agreement = 0.91, *p* < 0.05) (Koo and Li 2016). Even though providing a small sample, this example calls for a more detailed analysis which is not limited to such reliability indices, and provides further information. In the following, the ICC for agreement is used.

Therefore, the “absolute reliability” is often provided via the SEM. It should be noted that the SEM is just calculated by multiplication of the square root of 1 minus the ICC, with the standard deviation of the individual trial differences (Tighe et al. 2010):

3 $${\text{SEM}} = {\text{SD}}*\sqrt {1 - {\text{ICC}}} ,$$

where SEM is the standard error of measurement, SD is the standard deviation between trial 1 and trial 2, and ICC is the intraclass correlation coefficient.

In the concrete example mentioned above, this leads to ${\text{SEM}} = 2.97 {\text{cm}} \times \sqrt {1 - 0.93} = 0.79\;{\text{cm}}.$ Even though receiving a value in the same unit as the measurements, the close relationship to the ICC is obvious (Hopkins 2000; Weir 2005). Therefore, to counteract limitations of ICC based statistics Hopkins (2000) introduced calculating the typical error as follows:

4 $${\text{TE}} = \frac{{\mathop \sum \nolimits_{n = 1}^{i} {\text{SD}}\left( {x_{i} - y_{i} } \right)}}{\sqrt 2 },$$

where *n* is the number of data points, *i* is the index for each (paired) data point, *x*_*i*_ is the *i*-th data point in variable *x* (e.g., jumping height at trial 1), *y*_*i*_ is the *i*-th data point in variable *y* (e.g., jumping height at trial 2), and SD is the standard deviation of the mean differences between trial 1 and trial 2.

This would result in a higher error, with ${\text{TE}} = \frac{{3.78\;{\text{cm}}}}{\sqrt 2 } = 2.67\;{\text{cm}}.$

Aiming to consider the reliability to predict an increase that should be reached in a longitudinal testing design to improve interpretability of results, the minimal detectable change is often provided by multiplication of the SEM with 1.96 and the squared root of 2. Depending on the formula used to calculate the SEM , the MDC can be calculated by applying either the SEM or the TE in the following formula:

$${\text{MDC}} = {\text{SEM}}*1.96*\sqrt 2 ,$$

where MDC is the minimal detectable change and SEM is the standard error of measurement, resulting in a $MDC_{SEM} = 0.79 \times 1.96 \times \sqrt 2 = 2.18{\text{cm}}$ or, using the TE ${\text{MDC}}_{{{\text{TE}}}} = 2.67 \times 1.96 \times \sqrt 2 = 7,40\;{\text{cm}}$ for the jumping height testing example from five imaginary participants (Seamon et al. 2022). Obviously, this value is not only dependent on the ICC, but seems to provide limited advantages compared to the SEM.

A common limitation of interpretability of the TE (and of the SEM) is the reference value. Therefore, an appropriate way to state the TE or the SEM is to report the measurement error related to the mean (mean ± TE). However, in sports medicine and exercise research, heteroscedasticity of data must be taken into account (Nevill and Atkinson 1997), meaning that in individuals reaching the highest values in the test there would be a larger typical error expressed in units of the measurement (Bland and Altmann 1986; Nevill and Atkinson 1997; Nevill 1997; Giavarina 2015). To counteract, Hopkins (2000) suggested to state the CV of the TE:

$${\text{CV}}_{{{\text{TE}}}} = \frac{{{\text{TE}}}}{M} \times 100,$$

thus, $\frac{{2.67\;{\text{cm}}}}{33.7} \times 100 = 7.93\%$, where M is the mean of the mean of the two trials.

Even though the *r*_p_ and ICC indicate excellent reliability, which is confirmed by the SEM and MDC, it seems problematic that the TE was higher than the MDC. Furthermore, reviewing individual values, it gets obvious from a practical perspective that the second trial did not measure the same (or closely the same), as they partly differed with more than 20%. To account for these differences (and to determine a measure for actual agreement), the test–retest difference will be calculated. To account for different directions of this difference (±), this difference will be provided as an absolute value. This procedure is described in Willmott and Matsuura (2005), Kim and Kim (2016) and Sgayer et al. (2024) as appropriate to add a quantification of the mean random scattering around the systematic bias. The mean absolute error (MAE) is calculated by

$${\text{MAE}} = \frac{1}{n}*\mathop \sum \limits_{i = 1}^{n} \left| {x_{i} - y_{i} } \right|$$

where *n* is the number of data points, *i* is the index for each (paired) data point, *x*_*i*_ is the *i*-th data point in variable *x* (e.g., jumping height at trial 1), and *y*_*i*_ is the *i*-th data point in variable *y* (e.g., jumping height at trial 2).

Referring back to the previous jump testing example, this means the absolute of (40–35) + (45–49) + (36–31) + (32–29) + (22–18)/5 = 4.2 cm.

For a more intuitive interpretation as a percentage value, it can be expressed as the mean absolute percentage error (MAPE):

$${\text{MAPE}} = \frac{1}{n}*\mathop \sum \limits_{i = 1}^{n} \left| {\frac{{x_{i} - y_{i} }}{{x_{i} }}} \right|*100$$

where *n* is the number of data points, *i* is the index for each (paired) data point, *x*_*i*_ is the *i*-th data point in variable *x* (e.g., jumping height at time 1), and *y*_*i*_ is the *i*-th data point in variable *y* (e.g., jumping height at time 2).

Setting x_i_ as the first trial and y_i_ as the second trial results in absolute percentage errors of 14.2%, 8.16%, 16.13%, 10.3% and 22.2%, thus a MAPE of 14.23%.

In contrast, the CV results in smaller percentages compared to the MAPE, calculated via

$${\text{CV}} = \frac{{{\text{SD}}}}{{\overline{x}}}$$

where SD is the standard deviation between trial 1 and 2, and $\overline{x}$ is the mean between trial 1 and trial 2, which results in $\frac{{\frac{{\left( {3.54 + 2.83 + 3.54 + 2.12 + 2.85} \right)}}{5}}}{{\frac{{\left( {37,5 + 47 + 33,5 + 30,5 + 20} \right)}}{5}}}*100 = 8.81\% .$

### Data simulation to illustrate limited validity of relative reliability metrics to account for measurement errors

One could counteract that these limitations were simulated with an unrealistically small sample size, while most studies use larger sample sizes, therefore improving the validity of the ICC with an increasing number of participants, as individual deviations would account less. Therefore, in Table 1, a reasonable sample size of *n* = 40 is provided.

**Table 1:** Test–retest values of jumping heights with the respective mean and standard deviation (SD), level of significance from the *t* test for paired samples with the mean difference as well as the mean absolute error (MAE) and mean absolute percentage error (MAPE)

Participant	Test (in cm)	Retest (in cm)	MAE (in cm)	MAPE test–retest	MAPE retest–test
1	19	26	7	0.3684	0.2692
2	21	28	7	0.3333	0.2500
3	15	26	11	0.7333	0.4230
4	42	43	1	0.0238	0.0232
5	45	40	5	0.1111	0.1250
6	56	51	5	0.0892	0.0980
7	61	53	8	0.1311	0.1509
8	19	15	4	0.2105	0.2666
9	10	19	9	0.9000	0.4736
10	56	50	6	0.1071	0.1200
11	30	39	9	0.3000	0.2307
12	52	46	6	0.1153	0.1304
13	45	43	2	0.0444	0.0465
14	48	43	5	0.1041	0.1162
15	70	65	5	0.0714	0.0769
16	20	27	7	0.3500	0.2592
17	43	48	5	0.1162	0.1041
18	15	26	11	0.7333	0.4230
19	42	48	6	0.1428	0.1250
20	44	41	3	0.0681	0.0731
21	29	21	8	0.2758	0.3809
22	63	56	7	0.1111	0.1250
23	19	18	1	0.0526	0.0555
24	26	22	4	0.1538	0.1818
25	55	47	8	0.1454	0.1702
26	31	37	6	0.1935	0.1621
27	52	46	6	0.1153	0.1304
28	45	41	4	0.0888	0.0975
29	46	44	2	0.0434	0.0454
30	57	52	5	0.0877	0.0961
31	58	62	4	0.0689	0.0645
32	24	27	3	0.1250	0.1111
33	45	37	8	0.1777	0.2162
34	22	29	7	0.3181	0.2413
35	45	43	3	0.0444	0.0465
36	43	42	2	0.0232	0.0238
37	20	26	6	0.3000	0.2307
38	25	32	7	0.2800	0.2187
39	53	47	6	0.1132	0.1276
40	17	19	2	0.1176	0.1052
Mean	38.20	38.13	5.475	0.1972	0.1654
SD	16.23	12.67			
Systematic bias	Mean difference: 0.07 cm, *p* = 0.94				

#### Test–retest reliability

Calculating the bivariate Pearson correlation coefficient between test and retest resulted in a *r*_p_ = 0.94 while the corresponding ICC = 0.91, again, indicating excellent reliability (Koo and Li 2016). The corresponding SEM and MDC_SEM_ support this classification with 1.10 cm and 3.04 cm, respectively. The difference between the SEM and TE gets obvious, as the TE shows 4.32 cm (38.16 ± 4.32 cm) with a corresponding CV_TE_ = 38.16 ± 11.32%, while the CV was at 10.14%. Also with this data set, partially large individual scattering was shown (e.g., participant 7 shows a test–retest difference of 8 cm, which is equal to 13% with the first test value used as reference, participant 3 even shows a deviation of > 70%). Due to these limitations, the validity to consider these differences as excellent seems questionable.

**Fig. 1:**
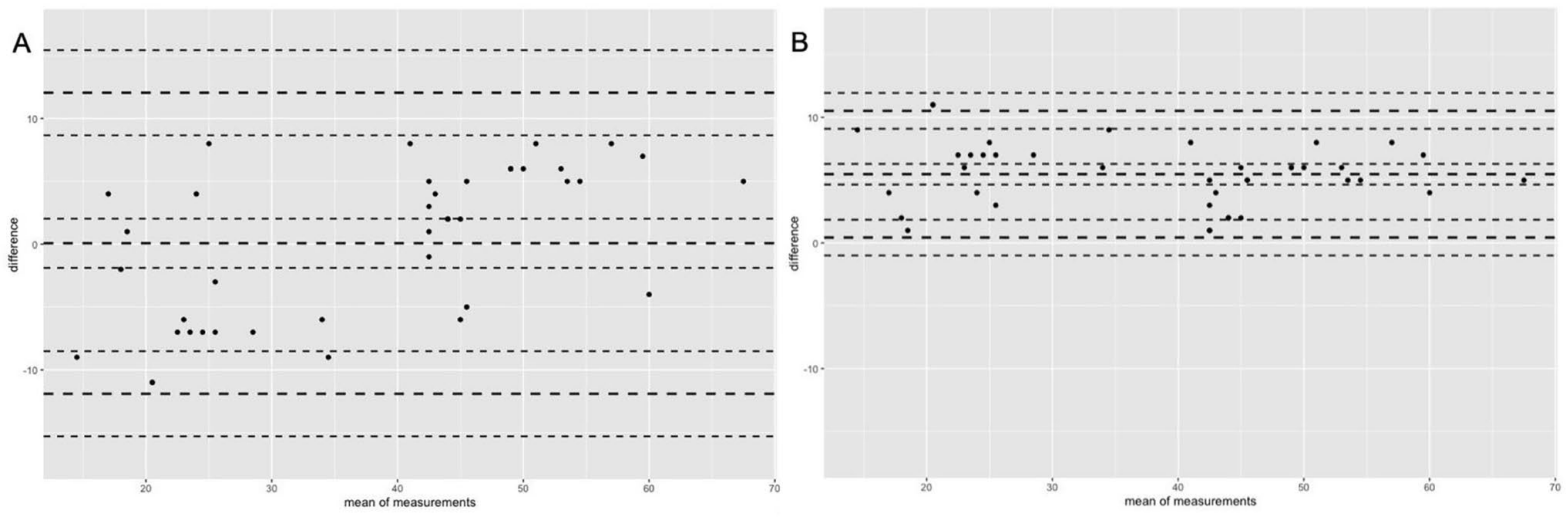
The BA plots for the same data set for test–retest (**A**) and re-organized data for best–second-best values (**B**)

According to the request from statistical papers to implement an agreement analysis such as Bland–Altman (BA) analyses (Bland and Altmann 1986; Kim and Lee 2022; Grgic et al. 2020) to reliability evaluations, such an analysis is provided in Fig. 1. In collaboration with a paired *t* test, the systematic bias between the means of the test and retest can be calculated and checked for significance. The graphical illustration in which the difference between two measures is plotted on the *y*-axis, while the mean of those is plotted on the *x*-axis. This allows a qualitative evaluation of the random scattering around the mean absolute error. The limits of agreement (LoA) contain 95% of those within the lower and upper limits.

With a mean difference of 0.07 cm and *p* = 0.94, the *t* test for paired samples indicated no considerable systematic bias (Atkinson and Nevill 1998), supporting the message of the ICC that testing was performed under reliable conditions. However, although excellent relative reliability and a lack of significant mean differences, the MAE shows a mean absolute deviation between the values of 5.48 cm with MAPE (dependent on the reference set to calculate the MAPE) between 16.5% and 19.7% with a maximal percentage error of 90% (Participant 9). LoAs ranged from − 11.9 to 12.05. In accordance with previous statements in the study, the larger sample size did not solve the problem and still resulted in a large random error (MAE/MAPE), although relative reliability was classified nearly perfect.

#### Re-organization of data: best–second best versus test–retest reliability

Another problem is the objectivity of the *r*_p_ and ICC, which can be reviewed in Table 2. Especially for studies that are interested in maximal performance changes, maximal values are considered for the further calculation and several tests were performed. Therefore, more than two trials are performed, limiting the possibility to perform test–retest reliability. To calculate the ICC, it is, however, possible to calculate the second-best–best reliability. By applying this simple sleight of hand trick, the relative reliability of one and the same data set can be even improved. In the following, the values from Table 1 were just re-arranged and the lower value was provided in column 1 and the higher value in column 2 to calculate the best–second-best reliability (Table 2).

**Table 2:** Second-best–best values with the respective mean and standard deviation (SD), level of significance from the *t* test for paired samples with the mean difference as well as the mean absolute error (MAE) and mean absolute percentage error (MAPE)

Participant	Second best (in cm)	Best (in cm)	MAE (in cm)	MAPE second-best–best	MAPE best–second best
1	19	26	7	0.3684	0.2692
2	21	28	7	0.3333	0.2500
3	15	26	11	0.7333	0.4230
4	42	43	1	0.0238	0.0232
5	40	45	5	0.1250	0.1111
6	51	56	5	0.0980	0.0892
7	53	61	8	0.1509	0.1311
8	15	19	4	0.2666	0.2105
9	10	19	9	0.9000	0.4736
10	50	56	6	0.1200	0.1071
11	30	39	9	0.3000	0.2307
12	46	52	6	0.1304	0.1153
13	43	45	2	0.0465	0.0444
14	43	48	5	0.1162	0.1041
15	65	70	5	0.0769	0.0714
16	20	27	7	0.3500	0.2592
17	43	48	5	0.1162	0.1041
18	15	26	11	0.7333	0.4230
19	42	48	6	0.1428	0.1250
20	41	44	3	0.0731	0.0681
21	21	29	8	0.3809	0.2758
22	56	63	7	0.1250	0.1111
23	18	19	1	0.0555	0.0526
24	22	26	4	0.1818	0.1538
25	47	55	8	0.1702	0.1454
26	31	37	6	0.1935	0.1621
27	46	52	6	0.1304	0.1153
28	41	45	4	0.0975	0.0888
29	44	46	2	0.0454	0.0434
30	52	57	5	0.0961	0.0877
31	58	62	4	0.0689	0.0645
32	24	27	3	0.1250	0.1111
33	37	45	8	0.2162	0.1777
34	22	29	7	0.3181	0.2413
35	43	45	3	0.0465	0.0444
36	42	43	2	0.0238	0.0232
37	20	26	6	0.3000	0.2307
38	25	32	7	0.2800	0.2187
39	47	53	6	0.1277	0.1132
40	17	19	2	0.1176	0.1052
Mean	35.43	40.90	5.475	0.2077	0.1550
SD	14.51	14.08			
Systematic bias	Mean difference: 5.48 cm, *p* < 0.001				

Relative reliability indices improved for *r*_p_ = 0.98 with an accompanied ICC of 0.92. This is, in terms of accuracy of special consideration as due to the re-organization of the data set, a significant systematic bias occurred (5.48 cm, *p* < 0.001). However, the MAE was robust for re-organization of the values and remained at 5.48 cm with only small, non-significant changes in the MAPE (20.8% and 15.5%). Due to changes in the systematic bias, the LoAs in the second example also changed to − 0.43 to − 10.52. Compared to the previous analysis, the re-organization caused a reduction of the total LoA span of about 50% (see Fig. 1) and the TE also decreased to 1.82 cm (before 4.32 cm).

## Interpretation

It seems invalid to exclusively focus on relative reliability, as these do not appropriately account for the systematic and random measurement error. In the presented jumping testing example, a systematic bias from two consecutive testing sessions (intraday) or performed on following days (interday) could indicate that participants were unfamiliar with the testing protocol, while a systematic increase suggests learning effects within the session or between sessions. This was, however, not reflected in the ICC. While in the first scenario, the results showed no significant systematic bias, in the second scenario, the r_p_ and ICC improved, and there was a significant systematic bias. Assuming the systematic bias would indicate, for example, learning effects. If there is a systematic bias, a lack of reliability would indicate an invalid measurement as not the jumping height, but the ability to learn the jump was the tested parameter. Accordingly, before using such a measurement in a pre–post-interventional study, habituation sessions are requested until the systematic bias becomes insignificant. Of note, especially in testing protocols in which repeating the test would cause fatigue (such as jumping and maximal strength), the systematic bias from trial to trial could disappear after several repetitions; however, it cannot be interpreted as sufficient learning. After several trials, fatiguing effects could counterbalance learning effects. Therefore, in specific, and especially coordinative demanding tests, the interday systematic error must be insignificant.

In contrast to the systematic bias, the random error refers to secondary variance. This might arise from daily fluctuations of fitness, influenced by factors such as recovery, fatigue, hydration and nutrition status (biological variability). Moreover, how participants arrived at the lab or if they have social stress etc. could impact the testing reliability. Even though this error cannot be completely prevented, it should be quantified to reasonably interpret results (International Organization of Standardization 2023). Therefore, the previous example demonstrates that first, although ICC indicates excellent reliability, a 20% mean absolute error with a maximum of up to 90% can occur. Second, just re-arranging the measurement values resulted in a meaningful decrease in the LoAs, which can indicate an increase in precision, what would be in accordance with an increase of the ICC. In addition, systematic error occurred, showing that the ICC does not account for systematic learning effects, while simultaneously the difference between precision and accuracy was illustrated: re-organizing the values implicated that the results were more precise as the LoAs decreased in their range meaningfully. However, due to systematic errors, accuracy was reduced, as it might be that learning effects and not the athletes maximal jumping height was actually tested here. In addition, an increase in precision derived from a reduction in the LoAs must be considered a misinterpretation, because the actual measurement error (MAPE) remained at 20%.

The authors and readers should, therefore, take great care when interpreting reliability quantifications exclusively reported via ICCs, as even seemingly excellent ICC values do not automatically imply accurate testing conditions. Further attention must be paid to the interpretation of the frequently recommended agreement analysis when seeking valid quantifications of measurement errors. To summarize, a detailed reliability analysis must include multiple measurement error quantifications that objectively (e.g., not vulnerable for data organization) account for systematic errors (learning effects, warm-up effects, fatiguing effects throughout the whole sample) and the random error/noise that stems, on the one hand from limitations and problems in the standardization, and on the other hand, from biological variability. An accurate interpretation is necessary, as the statistics alone do not distinguish which part of the secondary variance can be allocated to which source.

## Device versus testing reliability

These limitations underline the relevance to distinguish between device reliability and testing procedure reliability (Atkinson and Nevill 1998; Weakley et al. 2021). For instance, even though a force plate or contact mat measured ground-reaction force or flight time reliably (Tenelsen et al. 2019), this does not infer drop jumps a reliable test in all populations. However, it is common practice to refer to other studies or systematic reviews to justify a used testing protocol to be reliable. This problem is, however, not limited to jump testing, not even to sport science, but is a general problem that will be described via selected examples in the following. Without exploring reliability for the testing procedure, Gholipour Aghdam et al. (2024) referred to the Tegner activity score as a reliable test item with an ICC of 0.82. Wilke and Vogel (2020) and Niederer et al. (2019) referred to Galpin et al. (2008) to prove their testing procedure reliable (ICC = 0.89). Own reliability statistics are missing. Similarly, for the Stroop test, Wilke (2020) referred to Wöstmann et al. (2013) with an ICC of 0.81–0.86 for the trail making test (Wagner et al. 2011; Sanchez-Cubillo et al. 2009), while the digit span test was justified with *r*_p_ of 0.73 (Youngjohn et al. 1992) and did not explore the own testing procedure regarding reliability. Although there are numerous further examples, these articles underline common limitations in reliability interpretations that are based on a lack of knowledge regarding systematic and random measurement errors, as well as the content-related interpretations. The results of a narrative search on systematic reviews that recommend general clinical applicability based on pooling ICCs from original studies is provided in the supplemental material (see Table S2 in Supplemental Material). As the ICC does not account for the systematic, nor the random error, and it cannot be assumed that all participants are equally familiar with a testing protocol, nor secondary variance arise from equal sources in very different settings it does not seem appropriate to refer to systematic reviews to justify the own testing procedure reliable. Independent on chosen reliability metrics, this section must be differentiated from validity analyses. While for accurate testing, the device must be valid to assess the parameter, it does not automatically mean that even if the device measures valid (in which reliability is considered), it measures precisely, nor that in the respective population the device accuracy led to accurate testing results, as for this, protocol reliability and validity must be ensured.

## True value and reliability

Referring to reliability definitions “Reliability was originally defined as the ratio of true score variance to the observed total score variance in classical test theory (Cronbach et al. 1972; Lord and Novick 1986), and is interpreted as the percent of observed variance explained by the true score variance” (Barnhart et al. 2007). The underlying assumption is the availability of a true score. Even though the truth may exist, a true performance value (e.g., a “true” jumping height) seems more a theoretical construct, as this assumption neglects the biological variability of performance in human bodies. While, on the one hand, the repeatability can be considered the quantification of the ability to measure this truth or range of true values, measurement errors must be differentiated from normal biological intrasubject variance. While from a statistical perspective, daily form, fatigue, diet and more might be reflected as deviations from the test to retest, it is of high interest whether this secondary variance occurs due to a lack of standardization, or because of normal biological variance. For example, the muscle thickness of the gastrocnemius is measured via ultrasound imaging twice in a row, resulting in an outcome of 15 mm and 16 mm. It is unclear which measurement was performed adequately, and which not when referring to intraday reliability. If such a deviation of values is observed for interday, external factors such as previous activity could also have caused increased blood inflow to the muscle, and the 1 mm difference does not refer to measurement errors, but to normal biological variance. Atkinson and Nevill (1998) suggested using the TE as a measure for quantifying the borders of precision when determining true value, with serious limitations of including just about 68% of the values of the population. To accurately estimate the true value range and reduce the probability of mistakes, performing more trials will increase the likelihood that the mean will reflect the true muscle thickness in the chosen example. However, increasing the number of measurements will inevitably simultaneously increase the minimum and maximum obtained value, extending the borders of the measurable true muscle thickness.

Apart of errors in measurements and lack of standardization, variance in measurement results can result from variations in the true score. Coming back to the initially used example of jumping performance, daily form or fatigue, time of the day or, for instance, consumption of ergogenic substances such as caffeine consumption (Antonio et al. 2024) or others could influence the jumping performance systematically (if all participants are fatigued in the second test, e.g., after exhausting competition or training), or randomly (if, for example, not all participants consumed caffeine before the second test). This again underlines the relevance of distinguishing between precision and accuracy of testing. While the testing could be performed precisely (small random errors), it does not say anything about the accuracy, which would not just refer to hitting a point precisely, but hitting *the right point* precisely.

## The determination of limits of agreement

As outlined above, using the BA analysis is another valuable procedure to quantify reliability, if interpreted correctly. In a review on test–retest reliability of the one repetition maximum (1RM) strength measurement, Grgic et al. (2020) included 32 studies and outlined ICCs of 0.64–0.99 with a median of 0.97 and 92% of the ICCs ≥ 0.9, and a corresponding wide range of CVs between 0.5 and 12.1%. On the one hand, the authors support the request for advanced reliability indices without an exclusive focus on ICCs and CVs and suggested the inclusion of BA agreement analyses. Underlining the problem, only 7 out of 32 of the included studies provided LoAs. On the other hand, the authors of this review article reported LoAs for test–retest reliability in the 1RM strength test as follows: ± 3–5 kg, ± 5–8 kg, ± 8–13 kg, and ± 10–15 kg, making an interpretation impossible. Within an example of 20 kg as a mean value, a range of ± 10–15 kg seems very high, while this range can be reported neglectable with a reference mean of 500 kg. Therefore, lower and upper limits of agreement are exclusively interpretable with the corresponding reference values, or must be stated in percentage of the respective means depending on the evaluated construct.

LoAs can be easily interpreted incorrectly as the 95% CI. The LoA can be described as a reference interval or normal range for the test–retest differences expected for 95% of individuals, causing a probability statement for expected values (Wright and Royston 1999), while Atkinson and Nevill (2000) differentiated the 95% CI as follows: “the upper and lower boundaries within which a population parameter is thought to lie, given a particular statistic derived from a random sample. The most common coverage confidence for CI is also 0.95. The width of CI is dependent on sample size and the t-statistic is employed for calculation of inference to the population parameter (Atkinson and Nevill 2000).” Accordingly, Bland and Altman (1986) reported superficial similarity, however, no equality. Referring to underlying statistical approaches, a sample size of at least 50 should be included to appropriately interpret LoAs (Hopkins 2000; Bland and Altmann 1986; Bland and Altman 1999). Consequently, both the TE and the LoA describe a reference range. The LoA can be interpreted as a reference interval for test–retest variability, while the TE would express a reference interval for the true score error, which would actually cover approximately 68% of all true scores in the specific population (Atkinson and Nevill 2000; Harvill 1991). This difference is of crucial importance when reviewing the most frequent use of BA plots in today’s literature. Bland and Altmann (1986) originally used the BA plot to determine the statistical agreement between two clinical measurements (blood pressure devices) by referring to often inappropriately used relative reliability coefficients such as r_p_. Accordingly, similarly to other thresholds and borders, these limits must be set prior to the agreement calculation and should be determined by the context they are supposed to use for. Thus, Carstensen et al. (2008) described the LoAs as the range of expected values, if the procedure was repeated with the same participants under the same conditions. Even though statistically correct, this purely descriptive statement does not classify high or low LoAs as good or bad reliability. Indeed, which LoAs can be considered acceptable, thus indicate good reliability, strongly depends on the content and context, while simultaneously considering the “normal” variability attributable to, for instance, biological influencing factors. It seems of limited validity to state LoAs of measured values without describing a tolerable range for the anticipated use.

## Practical recommendations based on relative reliability

In accordance with previously published studies criticizing the use of Pearson correlation coefficients, the very limited validity of using *r*_p_ to evaluate reliability was confirmed in the data. Nevertheless, there are still numerous (recently published) studies that describe sufficient reliability based on correlation coefficients. Some examples are provided for the discussion.

Rhodes et al. (2022) included 30 male elite soccer players to evaluate the test–retest reliability of an isometric soleus muscle strength test. For the right and left legs, the means and standard deviations were stated as 1775.1 ± 486.7 N and 1733.9 ± 471.9 N, respectively, while in the retest, the authors measured 1846.27 ± 391.6 N in the right and 1767.6 ± 327.0 N in the left leg. Referring to r_*p*_ = 0.79–0.89, SEM of 161.41 and 216.24 N with SEM% of 9.09–12.47%, however, without providing the reference value. Interestingly, the calculated MDC, which was calculated with the previously mentioned formula by Ransom et al. (2020), is stated as 25.19 N and 34.56 N which is not verifiable on the basis of the data. It is impossible to receive smaller MDCs compared to the SEM ($161.41N*1.96*\sqrt 2$ cannot result in 25.19N or 34.56N MDC). Considering the quantification of the lower and upper limits of agreement with -494.76–352.49 N for the right and -591.30–523.82 N for the left leg and the qualitative inspection of the BA plots illustrating the random scattering around the (non-significant) systematic bias, this study is a perfectly fitting example for the previously mentioned limited interpretation of BA plots. The recommendation for the practical use to assess soleus muscle function via the assessed testing protocol is surprising from a practical and clinical perspective. Several further studies used *r*_p_ as a reliability quantification (e.g., Bollinger et al. 2020; Rosen et al. 2023; Plotnikoff and MacIntyre 2002), stating acceptable reliability with *r*_p_ ≥ 0.7.

Several studies do not only ignore the systematic and random measurement error, it seems that they post hoc classify almost every ICC as sufficient for the following result exploration. For example, Thomas et al. (2017) reported reliability of different tests with ICC ≥ 0.95 for horizontal jump testing, while strength testing showed values ICC ≥ 0.44. For Ripley et al. (2023), ICC > 0.61 (CV < 8.33%) was classified as fair to good reliability. Van der Made et al. (2021) performed isometric knee flexion tests in 30 male rugby players. The stated interpretation of ICCs ranging between 0.83 and 0.84 classified the testing procedure to be reliable. However, high ICCs can also be accompanied by large CVs. Wright et al. (2015) reported “almost perfect” reliability with ICC = 0.95 but CV of 9.3 and 34%. Other authors classified ICCs of 0.75 as acceptable (Mentiplay et al. 2015), 0.66 seems legitimate as well (Fraser et al. 2018). For Lam et al. (2014), ICCs of 0.49–0.98 with an interday reliability of 0.41 were fair enough to recommend Myoton measurements for the daily practical usage. Several studies referred to Atkinson and Nevill (1998) and McGraw and Wong (1996) when classifying their ICC ≥ 0.7 as reliable. For example, Martin-Rodriguez et al. (2017) and Wright et al. (2015) classified reliability of muscle assessments with *“*low (< 0.70), good (0.70–0.79), high (0.80–0.89), and excellent (≥ 0.90)”, quoting both listed studies. It is interesting, that Atkinson and Nevill (1998) did not recommend such a classification. In contrast, the authors criticized the focus on relative reliability and requested applying Bland–Altman analyses to examine agreement.

Other examples are provided from surveys and psychological studies. Psychometric scales are increasingly used in the clinician’s daily practice (Vetter and Schober 2018; Marasini et al. 2016). Lifland et al. (2018) underlined the importance of reliable measurement procedures in scientific work. However, certain ICCs ranged from 0.34 to 0.91 (Evensen 2003; Robinson et al. 2010; Yang et al. 2023), which are to be classified as insufficient although almost every accepted classification would indicate acceptable to excellent reliability. Performing a memory questionnaire in 101 participants at about 80 years of age reported ICCs of 0.72–0.77 (Yang et al. 2023). Without a systematic bias and means for the item’s satisfaction, ability and strategy, a mean score of about 40, 60 and 20 and BA plots revealed lower and upper limits of agreement, with -15.7 to 22.0, -24.2 to 24.7 and -21.4 to 21.3 points, respectively. Interestingly, while acknowledging intolerable high random error, the authors recommended the questionnaire “may be more appropriate for research purposes than for clinical purposes”, proofing a confusion science comprehension.

## Limitations

The objective of this work was to raise awareness and direct future data collections to take care of test–retest reliability quantification and the reporting of limitations. However, although necessary, future works must discuss content-related thresholds for reliability and, for example, the limits of agreement. Since, as described, these are content specific, much effort is necessary to determine device and test specific limits that can be considered acceptable. Accordingly, this must be done in separated works to guide future authors using, e.g., ultrasound muscle thickness determinations. In addition, how to exactly consider systematic and random testing errors to classify the certainty of evidence (which is already very common in meta-analysis by applying the GRADE working group criteria, which involves downgrading for heterogeneity and inconsistency in outcomes) must be explored and developed in future research. However, to account for and counteract limitations in current ways to calculate reliability, the authors must be aware of those to accept improved guidelines in the future. To solve this problem, one could suggest to take distance from sport-specific questions and develop more general statistical approaches. However, exactly this approach caused currently used “one-fits-all” reliability statistics without consideration of sport-specific/behavioral science related problematics. Although this paper illustrates a problem in the sports-specific setting, specific problems and limitations might also be present in other disciplines, which also should develop biostatistics that account for those specific circumstances, instead of adopting holistic and general classification approaches. Lastly, there are several further reliability metrics such as the SWC that were not considered in this article. Moreover, as there are several ways of calculating and reporting, for instance, the SEM, MDC or the SWC, a consensus on which reliability statistics must be implemented as standards is urgently needed to avoid confusion. Furthermore, it is so far not possible to distinguish between biological variability that is not avoidable in testing, but also cause secondary variance/random errors. Based on these, normal ranges of random errors specific for testing devices must be developed and it must be considered that the random error will never be 0.

## Conclusion

In general, it seems there is a substantial lack of awareness for (random) measurement errors, the way to evaluate these and consequences for the practical and clinical use. This work impressively illustrates that an ICC that is classified excellent can be accompanied with comparatively large measurement errors.

It is of paramount importance to, in a first step, quantify measurement errors, in a second step appropriately interpret the results (random error refers to standardization across the conducted tests) and in a third step to adjust the test protocol not just within one testing session, but throughout the complete test protocol. If an ICC can be considered acceptable or not is more a clinical decision rather than a statistical decision. It can be, therefore, summarized that for an adequate testing protocol, which include reliable, precise and accurate testing, a mean bias should not reach the level of significance, while simultaneously the random error must be minimized. Nevertheless, normal biological variance (such as influences of time of the day and variations in daily form) must be considered as normal secondary variance, which must be differentiated from measurement errors stemming from a lack of standardization.

## Outlook to future research designs including appropriate reliability reporting

**Fig. 2:**
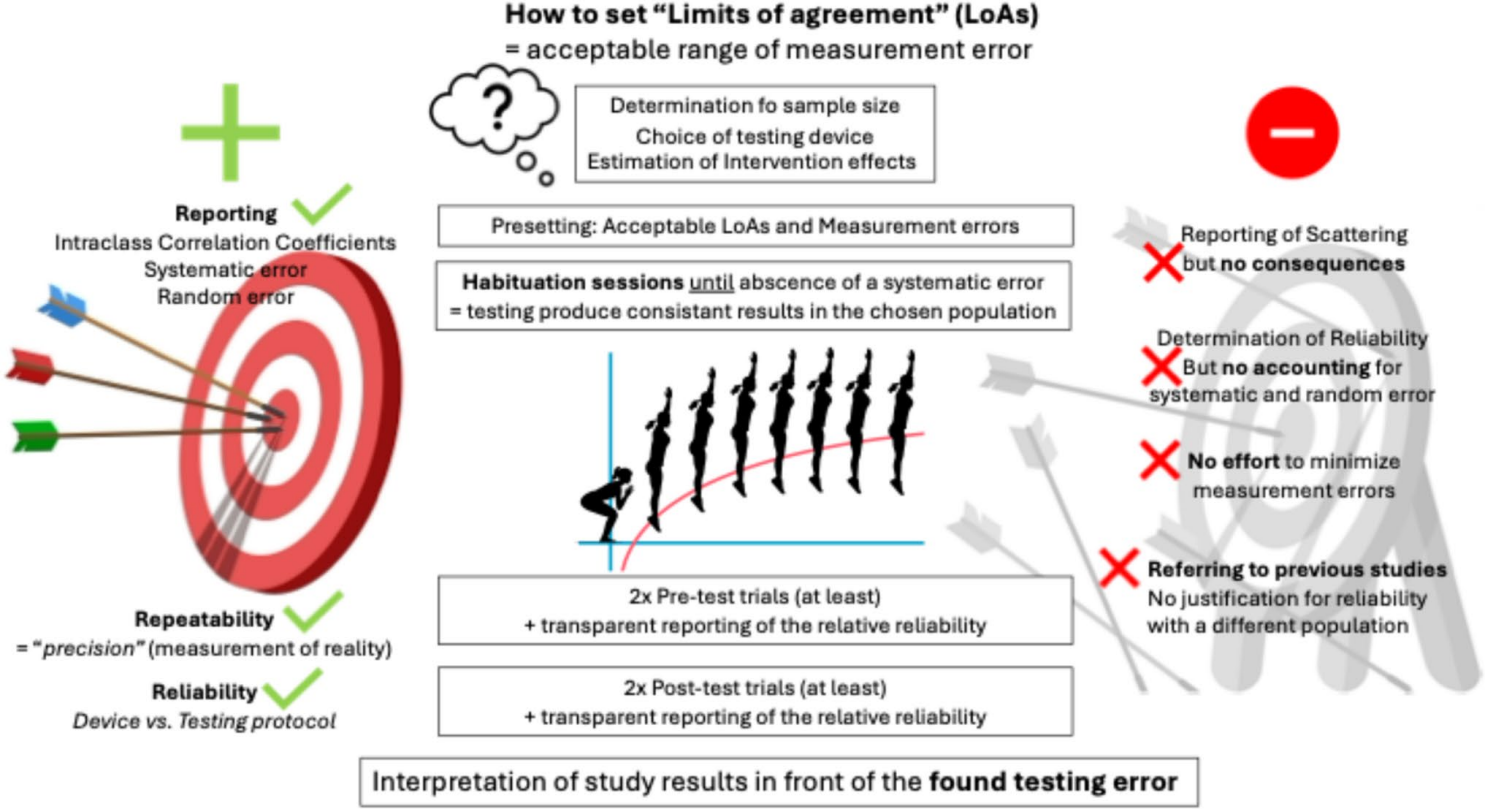
Our suggestion for research designs to improve reliability of data collections in empirical research with humans

Although guidelines to classify reliability metrics exist, all authors reported that reliability must be classified in the light of content and/or context. This proposal is, evidentially neglected by many authors in sports medicine and exercise science. Therefore, this work encourages future classifications of reliability in front of the content and context, in which the results must be interpreted. This request, however, is nothing new as provided by previous authors on reliability classifications (e.g., Bland and Altmann 1986; Koo and Li 2016; Cohen 1988). In addition to adequately account for different sources of variance in the test–retest agreement analytics, acceptable measurement errors and LoAs must be determined prior to the investigation and compared with the evaluated repeatability error in every individual population. If there are systematic errors (e.g., habituation effects) familiarization sessions are requested until intra- and interday reliability becomes minimal. Sources of secondary variance causing an increase in the random scattering should be avoided (e.g., experience of investigators with applied testing device and/or method procedure). Lastly, if a measurement error is acceptable, which LoAs can be tolerated must be determined in the light of the expected pre–post-change in longitudinal research designs. A proposed procedure to minimize the systematic and random error while transparently reporting measurement errors to meet high scientific standards is provided in Fig. 2. Therefore, if a reliability coefficient is considered sufficient, it crucially depends on expected pre–post-changes with pre-determined acceptable LoAs. Reliability must be controlled in every data collection for the individual population as it is not only appropriate to transfer device reliability, but not test reliability from trial to trial and population to population.

## Supplementary Information

Below is the link to the electronic supplementary material.

Supplementary file1 (DOCX 39 kb)

## Data Availability

Not applicable.

## Abbreviations

BA: Bland–Altman plot/analysis
CV: Variability coefficient/coefficient of variance
ICC: Intraclass correlation coefficient
LoA: Limits of agreement
MAE: Mean absolute error
MAPE: Mean absolute percentage error
MDC: Minimal detectable change
MS: Mean square
*n*: Number of participants
*r*_p_: Pearson correlation coefficient
SEM: Standard error of measurement
TE: Typical error
