# Supplementary material for: Discussion on the validity of commonly used reliability indices in sports medicine and exercise science: a critical review with data simulations
Source: Eur J Appl Physiol. 2025 Feb 13;125(6):1511–26. doi: 10.1007/s00421-025-05720-6 (PMC12174282; doi:10.1007/s00421-025-05720-6)
Supplement: Supplementary file 1 — Supplementary file1 (DOCX 39 kb) [file 421_2025_5720_MOESM1_ESM.docx]

**Supplemental Material**

**Table S1.** Definitions and terms as well as the calculation used in the literature.

| **Term** | **Definition** | **Calculation** |
| --- | --- | --- |
| Intraday/intrasession reliability | Measuring one parameter at least twice within one testing session | Mostly via ICC, SEM and MDC, CV |
| Interday/intersession reliability | Measuring one parameter at least twice in two separated sessions | Mostly via ICC, SEM and MDC, CV |
| Interrater reliability/objectivity | Measuring one parameter within one testing session by two independent tester | Mostly via ICC, SEM and MDC, sometimes quantified via r_p_, CV |
| Validity | Agreement between the gold standard device/procedure and the measuring device/procedure that should be validated | Mostly via ICC, SEM and MDC, sometimes quantified via r_p_, CV |
| Reproducibility | Measuring one parameter under same conditions in different populations, or measuring the parameter under different conditions in the same population | If performed via ICC, SEM and MDC, Sometimes quantified via r_p_, CV |
| Repeatability | Measuring one parameter under same conditions in the same conditions | If performed via ICC, SEM and MDC, Sometimes quantified via r_p_, CV |
| Precision | Multiple measurements of the same parameter should result in the same testing value, without considering whether the testing value was targeted | Can be quantified via random error analysis (e.g., Bland-Altman analysis, MAE, MAPE) |
| Accuracy | Multiple measurements of the same parameter should result in the same testing value, but considering that the testing value was targeted | No systematic error, minimal random error (within the ranges of biological variability of the parameter) |
| Agreement | Two tests (independent on repeated measures within one tester, between two testers or devices) should result in the same value | Correlation coefficients (Pearson or Spearman), Bland-Altman analysis, ICC for agreement |
| Internal consistency | Variability between repeated trials within a day (Intraday) | ICC |
| Stability | Equal to repeatability | See above |

**Table S2.** Selected review articles on testing reliability in different topics.

| **Author** | **Topic** | **Sample** | **Measures** | **Recommendation of the test** |
| --- | --- | --- | --- | --- |
| Powden et al., 2019 | Star excursion balance test and Y-balance test | N=9 | Intra rater: ICC=0.84 – 0.94, SEM=0.23 – 4.68  Reliability: good (one study moderate)  Inter rater: ICC=0.80 – 0.96, SEM=0.70 – 3.68  Reliability: good (one study moderate (although some studies lack SEM/MDC) | Excellent inter- and intra-rater reliability of the SEBT/YBT. SEBT/YBT should be used clinically to assess dynamic balance and provide consistent and repeatable results between one or more clinicians  **Recommendation for clinical practice** |
| Munoz-Berjmejo et al., 2021 | Five times sit to stand test in adults | N=8 | ICC=0.740 – 995  Good to excellent reliability | The FTSST is a very reliable measure of sitting and standing performance and assessment of lower limbs muscle strength and balance, regardless of whether individuals are healthy or suffering from any disease  **Recommendation for practice** |
| Rabelo et al., 2016 | Muscle strength assessment in chronic post-stroke hemiparesis patients | N=8 | ICC=0.48 – 0.99, for knee extension (pooled ICCs from 0.89 – 0.97), knee flexion (pooled ICCs from 0.84 – 0.91), plantar flexors (pooled ICC=0.85) | Objective muscle strength assessment can be reliably used in lower and upper extremities in post-stroke patients with chronic hemiparesis.  **Recommendation for practice** |
| Selistre et al., 2021 | Clinical Tests to measure strength and endurance in cervical muscles, handheld dynamometer | N=31 | ICC=0.64 – 0.9  Moderate – good intra and interrater reliability | Acceptable interrater and intrarater reliability  **Recommendation for practice** |
| Munoz-Bermejo et al., 2019 | Maximum strength measurements in isokinetic knee strength measurements in children | N=10 | ICC concentric extension: 0.50 – 0.98, SEM (from 6 studies: 5.5 – 16.3, %SEM=5.2 – 12.4, SRD (in 4 studies): 12.6 – 15.5, %MDC=14.4 – 30.9%  ICC concentric flexion: 0.31 – 0.96, SEM (from 6 studies: 3.1 – 25.0, %SEM=8.1 – 17.3%, MDC (from 4 studies): 8.5 – 13.0, %MDC=22.3 – 38.5%  Eccentric knee exteion: ICC=0.70 – 0.92, eccentric knee flexion: 0.60 – 0.86 | Isokinetic knee force measurement was reliable  **Recommendation for practice (also in children)** |
| Morral-Yepes et al., 2022 | Agility testing batteries in team sports | N=37 | ICC=0.79 – 0.99 (with expection of 3 studies)  Decision time ICC=0.95, movement time ICC=0.92, decision accuracy ICC=0.74 – 0.93 | Good reliability  **Recommendation for practice** |
| Bohannon, 2017 | Dynamometry based handgrip muscle | N=17 | ICC rated as good to excellent with >0.8  SEM more variable, MDC ranged from 14.5 – 98.5% | “Clinicians can be confident in the relative reliability of grip strength measures obtained from at risk older adults. However, relatively large percentage changes in grip strength may be necessary to conclude with confidence that a real change has occurred over time in some populations.”  **Recommendation for clinicians** |
| Garcia-Bernal et al., 2021 | Myotonometry for clinical application | N=5 | ICC:  0.75 – 0.82 excellent  0.65 – 0.93 moderate to high  0.79 – 0.96 excellent  0.75 – 0.96 high to very high  3 studies included LoA | Valid and reliable complementary to assess muscle viscoelastic properties in stroke survivors,  **Recommendation for clinicians** |
| Plisky et al., 2021 | Y-Balance test lower quarter | N=9 | Intrarater reliability: ICC=0.57 – 0.82 for adolscents and 0.85 – 0.91 in adults  Interrater reliability: ICC=0.81 – 1.0, test-retest reliability from five studies: 0.63 – 0.93 | YBT as a reliable tool for capturing dynamic single leg neuromuscular control  **Recommendation for practice** |
| Bonazza et al., 2017 | Injury predictive values of the functional movement screen | N=11 | Intrarater: ICC=0.81 (0.69 – 0.92 95% CI)  Interrater: ICC=0.81 (0.70 – 0.92 95% CI) | Excellent interrater and intrarater reliability.  **Recommendation for practice** |
| Clark et al., 2018 | Wii balance board to measure standing balance | N=25 | ICC = 0.27-0.997  MDC = 21-44%  Validity also measured | The WBB can provide data that is concurrently valid with typical commercial force platforms, and has reliability characteristics similar to force platforms for static standing computerised posturography.  **Recommendation for practice** |
| Reyes-Farrada et al., 2022 | Trunk strength measurement with isokinetic Dynamometer in non-specific low-back pain patients | N=5 | ICC=0.94-0.98 | The reliability of trunk strength assessment using an isokinetic dynamometer is excellent in patients with low back pain.  **Recommendation for practice** |
| Grgic et al., 2019 | Test – retest reliability of the Yo-Yo test | N=19 | ICC test – retest reliability = 0.78-0.98  CV = > 10% | The results of this review indicate that the Yo-Yo test has good-to-excellent test–retest reliability. These findings seem consistent regardless of the variant of the test and of the participants’ prior familiarization with the test.  **Recommendation for practice** |
| Thoris & Childs, 2018 | Rater reliability of Achilles tTndon size using ultrasound measurements | N=21 | Inter rater reliability ICC = 0.68-0.99  ICC Range: 0.26 - 0.99 | High risk of methodological bias; future reliability studies should use existing guidelines and develop existing guidelines to include elements of ultrasound imaging |
| Hanzlikova & Hebert-Loiser, 2020 | Landing Error Scoring System reliability | N=10 | ICC= 0.81-0.99  Interrater, intra rater and intersession reliability | LESS score has good-to-excellent intrarater, interrater, and intersession reliabilities.  **Recommendation for practice** |
| Hu et al., 2018 | Static and dynamic barefoot impression measurement reliability and identification | N=11 | Reel method and footprint angle ICC interrater reliability = 0.99  ICC range = 0.74 - 0.99 | Overall methodological quality was rated as ‘Poor’ to ‘Fair’.  **Recommendation for practice** |
| Keogh et al., 2019 | Smartphone applications to measure joint ROM | N=37 | ICC ≥ 0,75 | Weaker for absolute compared to relative measures such as ICC. Support the use of smartphones and apps in place of goniometers to measure joint motion  **Recommendation for practice** |
